# Apple consumption is associated with obesity- and lipid-related parameters and gut microbiota profiles across enterotypes: 12-week single-blind trial in Japanese adults

**DOI:** 10.3389/fnut.2026.1797920

**Published:** 2026-04-10

**Authors:** Toshihiko Shoji, Shiori Aoki, Yuki Sato, Mina Obara, Kazuaki Yoshinaga, Yoshiharu Takayama, Risa Araki, Saeko Masumoto, Tomisato Miura

**Affiliations:** 1 Institute of Food Research, National Agriculture and Food Research Organization, Tsukuba, Japan; 2 Institute of Radiation Emergency Medicine, Hirosaki University, Hirosaki, Japan; 3 Faculty of Food and Agricultural Sciences, Fukushima University, Fukushima, Japan

**Keywords:** (poly)phenol, apple, dietary fiber, gut microbiota enterotype, MaAsLin 3

## Abstract

Apples are rich in (poly)phenols and dietary fiber and have been associated with reducing the risk of chronic diseases. Emerging evidence suggests that inter-individual variability in metabolic responses to foods may be partly explained by differences in gut microbiota composition. However, whether apple induced metabolic responses differ according to baseline enterotype remains unclear. In this 12-week single-blind intervention trial, we investigated enterotype-stratified associations between daily apple consumption, gut microbiota profiles, and metabolic parameters in Japanese adults aged 40–65 years. Participants were classified into three enterotypes based on family-level gut microbiota composition; *Bacteroidaceae* (ET1), *Ruminococcaceae* (ET2) and *Prevotellaceae* (ET3). Multivariable association analysis using MaAsLin 3 identified five microbial genera associated with obesity and hyperlipidemia status after adjustment for repeated measures and relevant covariates, including *Bifidobacterium*, *Lachnospira*, *Prevotella*, *Anaerostipes*, and *Dialister*. While systemic lipid- or glucose-related host parameters did not differ across enterotype, fecal short-chain fatty acid concentrations increased significantly following apple consumption in participants classified as the *Bacteroidaceae*-dominant enterotype ET1. These findings suggest that baseline gut microbiota structures may modulate specific functional responses to apple intake, highlighting enterotype-dependent heterogeneity in metabolic adaptation.

## Introduction

The prevalence of chronic diseases such as obesity, type 2 diabetes (T2D), and cardiovascular disease (CVD) has significantly surged in both developed and developing countries (1, 2). Recent findings have been growing recognition of the intimate relationship between the gut microbiota and biological regulation, encompassing aspects such as host energy management and nutrient absorption. Even though how the gut microbiota affects host lipid metabolism is not fully understood, it is increasingly implicated as a crucial contributor to cardiometabolic disorders, including obesity, T2D, and metabolic syndrome. Human microbiome research has dramatically advanced with the practical use of next-generation sequencing. However, there is considerable individual variation in the gut microbiota and is influenced by nationality, diet, gender, body mass index (BMI) and age (3, 4). These variations make it difficult to demonstrate a clear relationship between the human microbiome and health. To address this challenge, recent studies have introduced the categorization, which classifies complex human gut microbial communities into the distinct gut types based on the dominance of specific genera (5).

Fruit consumption is associated with reducing the risk of chronic diseases including obesity and CVD (6, 7) and fruit (poly)phenols and dietary fiber contribute to these health benefits and are associated with reducing the risk of developing chronic diseases (8, 9). Apples (*Malus domestica* L.) are one of the most widely consumed fruits in the world. Apples are a rich source of several nutrients, including water-soluble dietary fiber, pectin, and a range of (poly)phenols (10). Several *in vivo* animal studies have suggested that consumption of the functional compounds in apple was likely to prevent metabolic diseases by improving the gut microbiota and modulating the Firmicutes/Bacteroidetes ratio in the gut microbiota in obesity and diabetes mice models (11–13). Particularly, we have shown that flavan-3-ols and procyanidins, main (poly)phenols in apple, promote lower Firmicutes/Bacteroidetes ratio, and reduced obesity and diabetes in the mice models (11). However, due to discrepancies between murine and human gut microbiota, the results of these murine studies cannot be directly extrapolated to human. Various recent human clinical trials involving the consumption of apples, apple-derived (poly)phenols or dietary fiber have highlighted their health benefits and potential for prevention of chronic diseases, including obesity, hyperlipidemia, T2D, and CVD (14–17). Moreover, several comprehensive (18, 19) and systematic reviews (20–23) have detailed the effects of apple and apple derived (poly)phenols on metabolic health. Recently, the effect of the gut microbiota population on host energy metabolism is receiving much attention, especially involvement in the onset and progress of CVD. Nevertheless, some studies on the preventive effects of apple consumption on chronic diseases have not yielded clear results focused on the gut microbiota. Therefore, it remains unclear how apple including (poly)phenols or dietary fiber consumption affects the gut microbiota and improves glucose/lipid metabolisms, that may be caused by considerable individual variations observed in the human gut microbiota. However, no previous study has evaluated whether enterotype modifies the metabolic response to daily whole-apple consumption.

In addition to baseline associations between gut microbiota composition and metabolic phenotypes (24), accumulating evidence suggests that inter-individual variability in metabolic responses to dietary interventions may be partly mediated by differences in the gut microbiome (25–27). Previous studies of dietary fiber, whole grain, and polyphenol interventions have consistently reported heterogeneous metabolic responses among individuals, implicating microbial composition as a determinant of responsiveness (25, 26). Enterotypes have been extensively utilized to categorize gut microbial community composition and have been linked to habitual dietary patterns and baseline metabolic characteristics (3–5). However, these associations primarily reflect cross-sectional relationships between enterotype and metabolic phenotype. The potential of enterotypes to function as predictors of differential metabolic responses to dietary interventions, that is, as response modifiers rather than static phenotype markers, remains an area that has not been sufficiently investigated, particularly in the context of fruit interventions.

In the present study, we conducted a 12-week single-blind intervention trial to evaluate the impact of daily apple consumption on obesity- and lipid-related metabolic parameters in Japanese adults. Importantly, rather than considering enterotype solely as a marker of baseline metabolic phenotype, we hypothesized that baseline enterotype may modify metabolic responses to apple intake. To test this hypothesis, we applied microbiome multivariable association with linear models 3 (MaAsLin 3) incorporating interaction terms between enterotype and metabolic status, enabling evaluation of enterotype-dependent associations following dietary intervention.

## Materials and methods

### Research ethics and subjects

The study protocol was approved by the Human Study Ethics Committee of the National Agriculture and Food Research Organization (Permit Number 22-028A; October 18, 2022) and all experimental procedures were conducted in accordance with the guidelines of the Declaration of Helsinki. This study was registered with the University Hospital Medical Information Network Clinical Trials Registry (UMIN-CTR, Japan; number UMIN000052142).

All subjects were asked to complete a questionnaire about their physical activity and smoking habits and to provide a copy of their medical records to obtain basic medical information. Men and women between the ages of 40 and 65 years with a BMI of 18.5 to 30 kg/m^2^ who were residents of Hirosaki, Japan were recruited for the study without a prior diagnosis of metabolic or gastrointestinal diseases. After receiving a full explanation of this study, all potential study subjects provided written informed consent prior to participation, and screening measurements of their gut microbiota were performed 1–2 months before commencing the trial. The exclusion criteria included subjects with a significant disease history of heart failure, dyslipidemia, diabetes, and uncontrolled hypertension; those on medication influencing body fat and serum lipids; pregnant and breastfeeding women; subjects with a medical condition requiring active management; and persons who may show allergic symptoms to foods (especially rosacea fruits, such as apples and peaches). Thirty-nine subjects (14 females and 25 males) were initially enrolled in the study. Although some participants exhibited elevated lipid parameters at baseline, none were receiving medical treatment for dyslipidemia. Additionally, information regarding the use of antibiotics, probiotics, and prebiotic supplements was collected at screening. Individuals reporting current use were excluded, and participants were instructed not to initiate such products during the intervention period.

### Study design

This study was a 12-weeks single-blind intervention trial with assessor blinding between January and April 2023. Laboratory personnel and outcome assessors were blinded to participant information and sampling time points during biochemical and microbiota analyses. Data analysts were also blinded to participant identity during statistical analysis. The subjects consumed one fresh “Fuji” apple (300 g) per day, peeled and cored. During the study period, subjects were instructed to maintain the same lifestyle (including diet and exercise) as before study participation. They were instructed to refrain from consuming supplements rich in (poly)phenols such as green tea and grape seeds. We also assessed the dietary intake of the subjects using a simple Food Frequency Questionnaire (FFQ) (28), which was developed for the Japan Public Health Center-based prospective Study (JPHC-NEXT). Food and nutrient intake were calculated on designed computer software (FFQ-NEXT, Kenpakusha, Tokyo, Japan) based on Standard Tables of Food Composition in Japan 2020 (8th revision) by the Ministry of Education, Culture, Sports, Science and Technology, the Government. Additionally, polyphenol intake was calculated by employing the food composition tables provided of Japan Standard Tables of Food Composition in conjunction with the National Agriculture and Food Research Organization (NARO) polyphenol database (29, 30). The intervention apple intake was recorded daily. Furthermore, the defecation frequency and Bristol stool form scale were assessed by the subjects on a daily basis (31).

### Intervention products

Fresh apples (“Fuji” variety) grown in Hirosaki, Aomori Prefecture, Japan were used as the intervention food. All apples were obtained from the Tsugaru Hirosaki Agricultural Cooperative Association (JA Tsugaru Hirosaki). All apples were directly delivered to subjects from JA Tsugaru Hirosaki on five separate occasions between January and April 2023. After delivery, the apples were stored in the dark under cold conditions for each subject. Clear instructions were given to eat the apple flesh, and not to consume the apple skin and core. They were not restricted to consuming apples, apple juice, or food made from apples.

The nutritional components of the intervention apples are listed in Supplementary Table 1. The (poly)phenol content was measured according to described methods (32, 33). According to our measurements, the amount of (poly)phenols presented in 300 g of apples consumed during the intervention period was 250.4 mg (the sum of the individual phenolic compounds). Particularly, a daily dose of 300 g “Fuji” apple contained 176.7 mg of procyanidins, accounting for 70.3% of the total (poly)phenol. Additionally, the amount of soluble and insoluble dietary fiber in one apple (300 g) was 1.2 g and 3.0 g, respectively, according to the Standard Tables of Food Composition in Japan (8th revision).

### Analysis of body mass index and visceral fat area

Physical examinations (including anthropometric and laboratory measurements) were performed before (baseline) and after each intervention (weeks 4, 8, and 12). The height and body weight of all subjects were measured. BMI was defined as weight (kg) divided by the square of the height (m). Waist circumference was measured at the midpoint between the lower thoracic border and iliac crest, and visceral fat area (VFA) was measured using an EW-FA90 visceral fat analyzer (Panasonic Corporation, Osaka, Japan) (34).

### Blood chemistry

Blood samples were collected from the peripheral veins of the subjects in the morning, after 12 h of overnight fasting. Clinical biochemical parameters were outsourced to LSI Medience Co. (Tokyo, Japan) according to the instructions of the vendors and comprised of total bilirubin and aspartate aminotransferase (AST), alanine aminotransferase (ALT), γ-glutamyl transpeptidase (γ-GTP), total cholesterol (TC), triglyceride (TG), total lipid, high-density lipoprotein cholesterol (HDLc), quantitative C-reactive protein (CRP), total bile acid, fasting plasma glucose (FPG), low-density lipoprotein cholesterol (LDLc), hemoglobin A1c (HbA1c), 1.5-anhydroglucitol (AG), alkaline phosphatase (ALP), and lactate dehydrogenase (LDH) levels.

### Short-chain fatty acid s analysis by gas chromatography-mass spectrometry

Each fecal sample was collected using a fecal collection kit with a small spoon (TechnoSuruga Laboratory Co., Ltd., Shizuoka, Japan) without preservatives. After the harvest, fecal samples were stored at −80 °C. Fecal short-chain fatty acid (SCFA) levels were measured using a modified version of the method reported by Garcia-Villalba et al. (35). Briefly, samples (approximately 100–200 mg) were weighed and placed in a bead tube. Phosphoric acid solution (0.9 mL of 0.5%) was added and grinded using a Precellys^®^ 24 tissue homogenizer at 5,500 rpm for 25 s. The sample was centrifuged (13,000 × g for 10 min at 5 °C), and 0.5 mL of the supernatant was transferred to a new microtube, mixed with an equal volume of diethyl ether, and 50 μL of 1 mM 2-ethyl butyric acid as an internal standard. After stirring for 10 min and centrifugation (13,000 × g for 10 min at 5 °C), the diethyl ether layer was transferred to a sample vial. The concentration of SCFAs (acetic acid, propionic acid, and butyric acid) in each sample was detected using a combined gas chromatograph-mass spectrometer (GC-MS) instrument (GC-MS QP2020NX, Shimadzu Corporation, Kyoto, Japan) with a InertCap Pure-Wax column (30 m × 0.25 mm, i.d. 0.25 μm, GL Science Inc., Tokyo, Japan). Helium was used as the carrier gas at 1.0 mL/min and an injector temperature of 250 °C. Injections were made in split (split ratio of 50:1) mode with an injection volume of 1 μL. The ionization source temperature was kept at 280 °C. The oven temperature program was as follows: 50 °C for 5 min; then 8 °C/min to a final temperature of 250 °C, and held for 1 min. The electron ionization (EI) scan range was *m*/*z* 40–450. The amounts of SCFAs were quantified by integrating the extracted ion chromatographic peaks for the following ion species: *m*/*z* 43 for acetic acid at 12.9 min, *m*/*z* 74 for propionic acid at 14.4 min, *m*/*z* 60 for butyric acid at 15.9 min, and *m*/*z* 88 for 2-ethyl butyric acid at 17.9 min. The absolute levels of SCFAs were quantified using calibration curves of individual SCFAs and 2-ethyl butyric acid.

Changes in bacterial genera were calculated as Log2 fold changes between post-intervention and baseline relative abundances with a pseudocount added to avoid zero values. Correlations between changes in bacterial genera and short-chain fatty acid levels were assessed within the *Bacteroidaceae*-dominant enterotype.

### Fecal DNA extraction and 16S rRNA gene sequencing

The DNA was extracted from the fecal sample using a NucleoSpin^®^ DNA Stool Kit (Macherey-Nagel GmbH, Düren, Germany) according to the manufacturer’s instruction. DNA concentration was measured using a NanoDrop Lite Spectrophotometer (Thermo Fisher Scientific, Wilmington, DE, United States), and the extracted DNA was stored at −30 °C until further analysis.

Sequencing and initial bioinformatic processing were outsourced to the Bioengineering Laboratory Co., Ltd. (Sagamihara, Kanagawa, Japan). Briefly, the V3–V4 region of the bacterial 16S rRNA gene was amplified using primers 341f_MIX (ACACTCTTTCCCTACACGACGCTCTTCCGATCT-NNNNN-CCTACGGGNGGCWGCAG) and 805r_MIX (GTGACTGGAGTTCAGACGTGTGCTCTTCCGATCT-NNNNNN-GACTACHVGGGTATCTAATCC). These primers consisted of an overhang adapter sequence required for the second PCR, 0–5 bases of random sequences (*N*) used for sequencing quality control, and gene-specific sequences for 16S rRNA amplification. The PCR amplification was performed in a 10 μL reaction mixture containing 1 μL of 10 × Ex buffer (TaKaRa Bio, Otsu, Shiga, Japan), 0.8 μL dNTPs (TaKaRa Bio), 0.5 μL each of the forward and reverse primers, 2 μL template DNA, and 0.1 μL of Ex Taq polymerase (TaKaRa Bio). Thermal cycling conditions were as followed: initial denaturation at 94 °C for 2 min; 30 cycles of 94 °C for 30 s, 60 °C for 30 s, and 72 °C for 30 s; followed by a final extension at 72 °C for 5 min. Amplified products were purified using an AMPure XP Beads Kit (Beckman Coulter, Inc., Brea, CA, United States) according to the manufacturer’s instructions, and the product quality was verified by agarose gel electrophoresis.

### Gut microbiome bioinformatics analysis

For next-generation sequencing analysis, the first PCR products were submitted to the second PCR using index-adapted primers to generate paired-end libraries (2 × 300 bp) and sequenced on the MiSeq platform (Illumina, Inc., San Diego, CA, United States) using the MiSeq Reagent Kit v3 (Illumina).

After sequencing, primer sequences were removed using the FASTX-Toolkit (version 0.0.14). Low quality sequences (quality score <20) were filtered using Sickle (version 1.33). Sequences <130 bases and their paired reads were discarded to remove extremely short reads that may result from sequencing artifacts or incomplete reads, while retaining sufficient sequence information for downstream amplicon processing. The paired-end reads were merged using FLASH (version 1.2.11) with the following parameters: merged sequence length of 410 bases, lead binding length of 280 bases, and a minimum overlap of 10 bases. The merged reads were subsequently processed as single-end reads using the DADA2 denoise-single pipeline implemented in the Quantitative Insights into Microbial Ecology version 2 (QIIME2, version 2024.2) (36) to perform quality filtering, denoising, chimera removal, and inference of amplicon sequence variants (ASVs). Representative ASV sequences were taxonomically assigned using the feature-classifier plugin against the Greengenes reference database (version 13_8) (37), which is clustered at the 97% sequence similarity. Downstream statistical analyses were performed at the genus level using relative abundance profiles derived from the ASV table.

### Biostatistics

Further analyses were conducted in the R programming environment (version 4.5.1) using RStudio. For the analysis of gut microbial community enterotypes, we employed Jensen–Shannon divergence (JSD) from the family-level relative abundance matrix and partitioning around medoid (PAM) clustering, which was performed using the cluster (version 2.1.8.1) package (3). The optimal number of clusters was set to three, with the maximum value of the Calinski–Harabasz (CH) index with the clusterSim package (version 0.51-5). Principal coordinate analysis (PCoA) was conducted using the dudi.pco function in the ade4 package (version 1.7-23).

Associations between gut microbiota features and obesity and lipid-related parameters were assessed using a multivariable association with linear models MaAsLin 3 package (version 1.1.2) (38). The longitudinal data obtained at baseline and weeks 4, 8, and 12 were analyzed using linear mixed-effects models, with subject identifier included as a random effect to account for repeated measurements within individuals.

Obesity status was defined based on BMI, with participants classified as obese if their BMI was ≥25 kg/m^2^, according with commonly used criteria following the guideline of the Japan Society for the Study of Obesity (JASSO) (39). Hyperlipidemia status was defined as a categorical variable based on serum lipid. Participants were classified as having hyperlipidemia if they met either of the following criteria following the guideline of the Japan Atherosclerosis Society (JAS) (40): TG concentration ≥150 mg/dL or LDLc concentration ≥140 mg/dL or HDLc concentration <40 mg/dL. Obesity and hyperlipidemia status were treated as time-varying categorical variables in longitudinal analyses. Gut microbial relative abundances at the genus-level were used as dependent variables. Microbial features were filtered prior to analysis. Genera with zero prevalence across all samples and those annotated as unclassified at the genus level were excluded. No additional abundance or prevalence thresholds were applied, and all remaining genera were retained for downstream analysis. Relative abundance data were normalized using total sum scaling (TSS) and log-transformed after the addition of a pseudo count. Time was modeled as a categorical fixed effect corresponding to study visit (baseline, weeks 4, 8, and 12), with baseline set as the reference level. This specification allowed for non-linear temporal changes and facilitated interpretation of interaction effects with enterotype.

For analyses of obesity status, Model 1 included serum TG levels as a time-varying covariate, together with time (study visited weeks), sex, and gut microbiota enterotype as fixed effects. The model was specified as:


$$\begin{array}{l} \text{Microbial feature}\sim (1|\text{Subject})+\mathrm{Sex}+\mathrm{TG}+\text{Time}+\text{Obesity}\\ +\text{Enterotype}+\text{Obesity}:\text{Enterotype} \end{array}$$

Model 2 was a corresponding main-effect model without the interaction term:


$$\begin{array}{l} \text{Microbial feature}\sim (1|\text{Subject})+\mathrm{Sex}+\mathrm{TG}+\text{Time}\\ +\text{Obesity}+\text{Enterotype} \end{array}$$

Similarly, for analyses of hyperlipidemia status, Model 3 included BMI as a time-varying covariate, together with time (study visited weeks), sex, and gut microbiota enterotype as fixed effects. The model was specified as:


$$\begin{array}{l} \text{Microbial feature}\sim (1|\text{Subject})+\mathrm{Sex}+\mathrm{BMI}+\text{Time}\\ +\text{Hyperlipidemia}+\text{Enterotype}+\text{Hyperlipidemia}:\text{Enterotype} \end{array}$$

Model 4 was a corresponding main-effect model without the interaction term:


$$\begin{array}{l} \text{Microbial feature}\sim (1|\text{Subject})+\mathrm{Sex}+\mathrm{BMI}+\text{Time}\\ +\text{Hyperlipidemia}+\text{Enterotype} \end{array}$$

In addition, sensitivity analyses were performed using genus-level enterotype classification (Models 5 and 6) to evaluate the robustness of the observed associations. The model was specified as, respectively:


$$\begin{array}{l} \text{Microbial feature}\sim (1|\text{Subject})+\mathrm{Sex}+\mathrm{TG}+\text{time}\\ +\text{Obesity}+\text{Enterotype}\;(\text{genus}-\text{level})\\ +\text{Obesity}:\text{Enterotype}\;(\text{genus}-\text{level}) \end{array}$$

$$\begin{array}{l} \text{Microbial feature}\sim (1|\text{Subject})+\mathrm{Sex}+\mathrm{BMI}+\text{Time}\\ +\text{Hyperlipidemia}+\text{Enterotype}\;(\text{genus}-\text{level})\\ +\text{Hyperlipidemia}:\text{Enterotype}\;(\text{genus}-\text{level}) \end{array}$$

Multiple testing correction was performed using the Benjamini–Hochberg false discovery rate (FDR) procedure, and associations with an adjusted *q* value <0.05 were considered statistically significant.

### Descriptive and inferential statistics

Data are presented as means with standard deviation. Data were analyzed using GraphPad Prism^®^ version 10 for Macintosh software. Paired *t*-tests were used to compare differences in lipid metabolism related parameters and SCFAs within each enterotype before and after the intervention. Between-group differences were evaluated using one-way ANOVA with Tukey’s multiple comparison test. For analyses involving multiple comparison, *p*-values were adjusted using the Benjamini–Hochberg FDR procedure, and the resulting adjusted *p*-values are reported as *q* values. Differences in dietary intake before and after the intervention were assessed using paired *t*-tests. All results were considered statistically significant at *p* < 0.05.

## Results

### Study population characteristics, dietary intakes, and compliance

**Figure 1:**
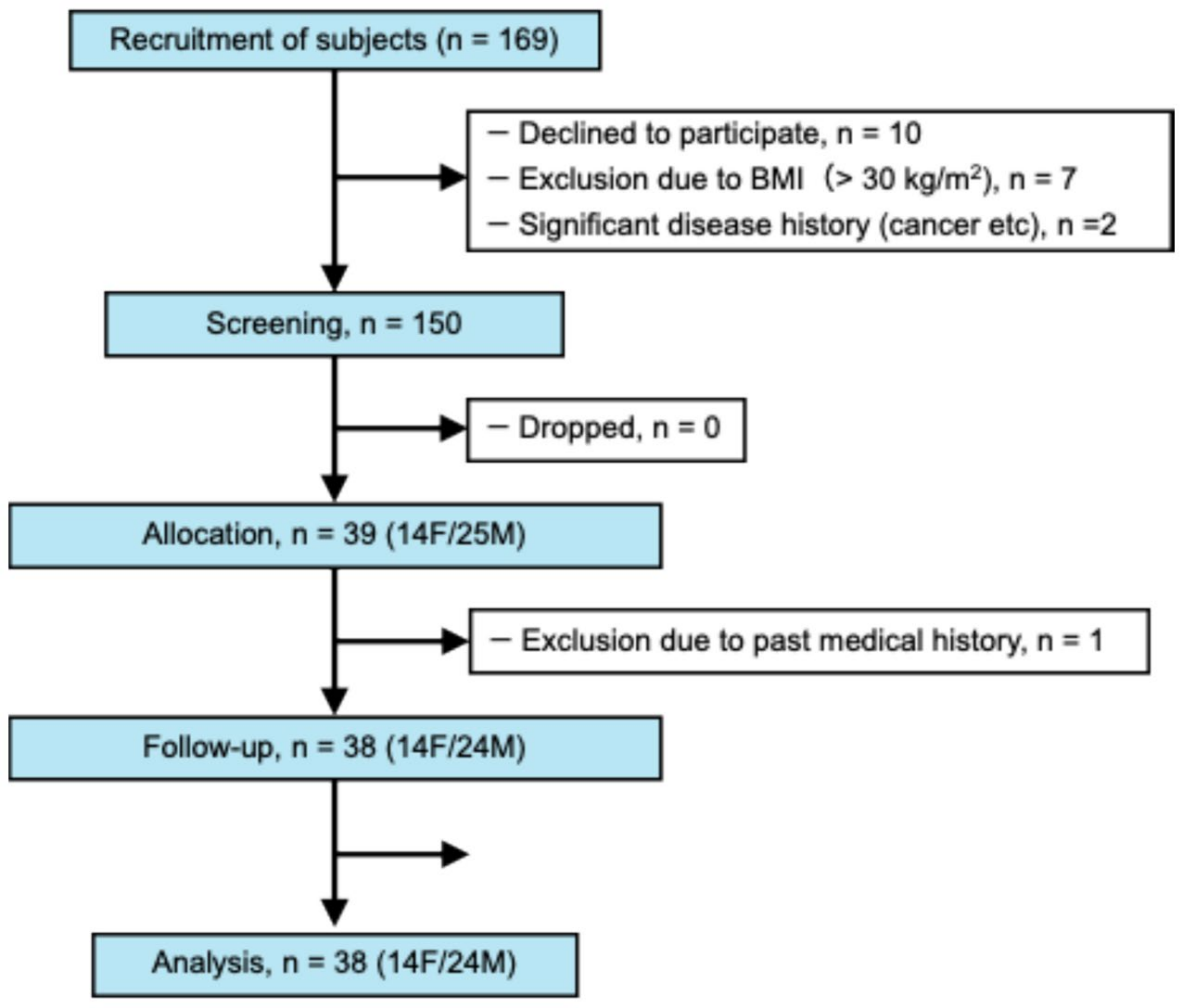
Flowchart of the experimental design for the study.

A subject flow diagram of the study is shown in Figure 1. Recruitment for the study was concluded by November 2022, and 39 patients were selected from the initial 150 who provided consent for participation. After allocation, one subject withdrew from the study owing to a history of intestinal disease. Thirty-eight subjects (24 males and 14 females) completed the 12-week intervention trial. In total, one subject who deviated from the protocol during the intervention period was excluded, and 38 subjects were finally included in the analysis.

The baseline date of anthropometric and clinical characteristics, blood biochemistry and habits in the females (*n* = 14) and males (*n* = 24) were shown in Supplementary Table 2. The age range of subjects was 41–63 years (mean: 51.7 years) and the BMI was between 18.5 and 29.6 kg/m^2^ (mean: 23.5). Additionally, we also estimated the energy and selected nutrients of the subjects using a simple FFQ at 0 week (baseline) and 12-week (Supplementary Table 3). At the baseline and end of the study, no significant differences were observed in the daily intake of total energy, carbohydrates, lipids, protein, soluble dietary fiber, insoluble dietary fiber, and polyphenols throughout the study period. The daily intake of apples, the test food, by the subjects during the study period was confirmed using questionnaires, and the intake was 288.3 g per day, with a consumption rate of 98% among all subjects. Consequently, the nutrient yield from the test food of the apple intervention was determined to be 152.8 kcal for total energy, 44.7 g for carbohydrates, 4.0 g for dietary fiber, and 240.6 mg for polyphenols.

### Baseline gut microbiota composition

A total of 6,964 ASVs were identified, comprising 10 phyla and 82 genera. Fecal bacteria at the baseline were dominated by Firmicutes (56.4 ± 10.4%), Bacteroidetes (29.9 ± 9.13%), Actinobacteria (10.7 ± 6.96%), Proteobacteria (2.35 ± 4.37%), and Verrucomicrobia (0.56 ± 1.30%). Fifteen genera were identified in more than 90% of subjects at the baseline. The genera with more than 1% of the total relative abundance were *Bacteroides* (21.0 ± 8.90%), *Faecalibacterium* (9.92 ± 6.52%), *Blautia* (9.24 ± 5.01%), *Bifidobacterium* (7.07 ± 6.44%), *Coprococcus* (5.17 ± 3.29%), *Roseburia* (4.43 ± 4.05%), *Prevotella* (4.25 ± 10.22%), *Collinsella* (2.98 ± 3.89%), *Ruminococcus* (2.66 ± 2.72%), *Parabacteroides* (2.31 ± 2.21%), *Oscilospira* (1.57 ± 1.63%), *Megamonas* (1.61 ± 5.99%), *Phascolarctobacterium* (1.47 ± 1.95%), *Clostridium* (1.03 ± 0.91%), and *Escherichia* (1.15 ± 4.17%) genera at the baseline (Supplementary Figure 1).

### Classification of subjects according to the gut enterotype at the baseline

**Figure 2:**
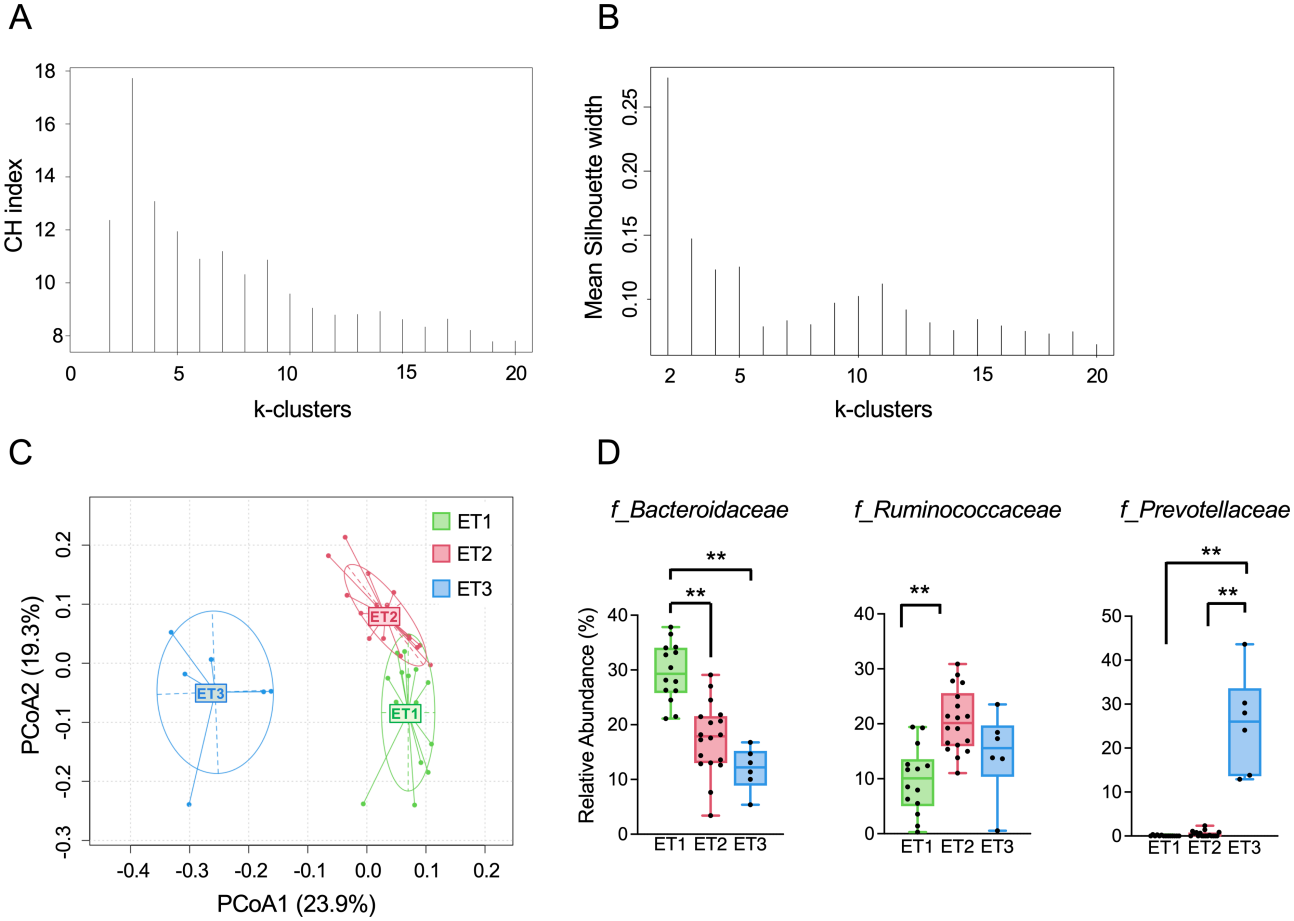
Gut microbiota enterotypes identified at baseline based on family-level relative abundance. **(A)** Calinski–Harabasz (CH) index for determining the optimal number of clusters. **(B)** Mean silhouette width values for each candidate cluster solution. **(C)** PCoA of family-level relative abundance clustered using PAM, showing three enterotypes: *Bacteroidaceae* (ET1), *Ruminococcaceae* (ET2), and *Prevotellaceae* (ET3). **(D)** Relative abundances of dominant families (*Bacteroidaceae*, *Ruminococcaceae*, and *Prevotellaceae*) across enterotypes. Boxes represent the interquartile range with median; whiskers indicate minimum and maximum values. ^*^p < 0.05 and ^**^p < 0.01 by multiple comparisons adjusted using the Benjamini–Hochberg FDR method.

In all subjects, there were no statistically considerable alterations in the anthropometric characteristics and blood biochemistry, including glucose and lipid metabolism markers by apple consumption. Next, to determine inter-individual variability of all subjects for a clearer understanding of the impact of apple consumption on gut microbiota, we assigned enterotypes of each subject and assessed a differential response of apple consumption on anthropometric characteristics and blood biochemical markers of the subjects. Clustering was performed based on the relative abundance at family-level using JSD distance and the PAM clustering algorithm. The optimal number of clusters was validated using the Calinski–Harabasz (CH) index and mean silhouette values. The CH index of gut microbiota was indicated an optimal solution of *k* = 3 (Figure 2A). Mean silhouette values were modest overall and slightly higher for *k* = 2, consistent with the continuous nature of gut microbiota variation. At baseline, subjects were categorized into three clusters (Figure 2B); *Bacteroidaceae* enterotype (ET1, *n* = 14), *Ruminococcaceae* enterotype (ET2, *n* = 18), and *Prevotellaceae* enterotype (ET3, *n* = 6) (Figure 2B). Family-level aggregation preserved a three-cluster structure consistent with canonical enterotypes. Furthermore, to assess robustness to taxonomic resolution, clustering was additionally performed at the genus-level (Supplementary Figure 2). Although genus-level clustering yielded a two-cluster solution with slightly higher silhouette values, this binary partition merged ET1 and ET2. Therefore, family-level classification was retained for subsequent analyses to preserve the observed three-cluster configuration.

In the *Bacteroidaceae*-dominant ET1 group, the relative abundance of *Bacteroidaceae* was significantly higher than other enterotypes, whereas those of *Ruminococcaceae* in the *Ruminococcaceae*-dominant ET2 group were significantly higher than that of ET1 (Figure 2C). The relative abundance of *Prevotellaceae* in ET3 group was significantly higher than other enterotypes. Moreover, at the phylum level, significant differences were observed between each gut enterotype group in the Bacteroidetes phylum at baseline (Table 1). The relative abundance of Firmicutes in the ET2 group was significantly higher than that of the ET1 and ET3 groups (*q* < 0.05). Additionally, the relative abundance of the genera *Bacteroides* in the ET1 group was significantly higher than that of the ET2 and ET3 groups (*q* < 0.05). The significantly differences were observed in the relative abundance of *Coprococcus* and *Faecalibacterium* genus in the ET1 group (Table 2).

**Table 1:** Relative abundance of bacterial phyla across gut microbiota enterotypes at baseline.

Phyla	Enterotype			
	ET1 (*n* = 14)	ET2 (*n* = 18)	ET3 (*n* = 6)	*q* value
Bacteroidetes				0.0004^**^
	33.4 ± 5.57	23.2 ± 5.29	41.8 ± 8.64	0.1437
				<0.0001^**^
Firmicutes				0.0023^**^
	50.9 ± 9.56	62.8 ± 8.20	50.2 ± 8.04	0.8502
				0.0093^**^
Actinobacteria				0.5665
	11.1 ± 7.76	12.0 ± 6.63	5.75 ± 4.10	0.1679
				0.1130
Proteobacteria				0.4397
	3.79 ± 6.87	1.37 ± 1.48	1.95 ± 1.28	0.4397
				0.3802
Verrucomicrobia				0.9830
	0.70 ± 1.77	0.52 ± 1.04	0.32 ± 0.77	0.9830
				0.9830

Values are presented means ± standard deviation.

*q* values were calculated using the Benjamini–Hochberg FDR method.

Pairwise comparisons were performed as follows: ET1 vs. ET2 (top), ET1 vs. ET3 (middle), and ET2 vs. ET3 (bottom).

Statistically significant differences are indicated by asterisks (^*^q < 0.05; ^**^q < 0.01).

**Table 2:** Relative abundance of bacterial genera across gut microbiota enterotypes at baseline.

Genera	Enterotype			
	ET1 (*n* = 14)	ET2 (*n* = 18)	ET3 (*n* = 6)	*q* value
*[Ruminococcus]*				0.4203
	2.26 ± 1.89	2.49 ± 1.63	2.80 ± 1.14	0.3996
				0.4203
*Bacteroides*				0.0002^**^
	29.6 ± 5.34	17.4 ± 6.48	11.9 ± 3.98	0.0001^**^
				0.1433
*Blautia*				0.2273
	9.45 ± 6.51	10.2 ± 4.10	6.02 ± 1.66	0.2273
				0.0868
*Bifidobacterium*				0.5109
	8.24 ± 6.21	7.50 ± 7.20	3.06 ± 2.52	0.2507
				0.2935
*Clostridium*				0.7713
	1.23 ± 1.12	1.00 ± 0.76	0.63 ± 0.78	0.2194
				0.2194
*Collinsella*				0.8444
	2.41 ± 3.03	3.70 ± 4.77	2.14 ± 2.73	0.8444
				0.8444
*Coprococcus*				0.0018^*^
	3.30 ± 3.15	6.81 ± 3.14	4.61 ± 1.14	0.2482
				0.2423
*Escherichia*				0.9755
	2.58 ± 6.72	0.28 ± 0.61	0.41 ± 0.89	0.9755
				0.9755
*Faecalibacterium*				0.0164^*^
	6.07 ± 5.43	12.6 ± 6.15	10.8 ± 6.39	0.1980
				0.5885
*Megamonas*				0.0886
	4.08 ± 9.52	0.21 ± 0.85	0.00 ± 0.00	0.0886
				0.5831
*Oscilospira*				0.3334
	1.23 ± 0.94	2.04 ± 2.13	0.95 ± 0.64	0.7353
				0.3334
*Parabacteroides*				0.9662
	2.60 ± 3.02	2.05 ± 1.29	2.43 ± 2.51	0.9662
				0.9662
*Prevotella*				0.2523
	0.06 ± 0.11	0.44 ± 0.66	25.5 ± 11.4	<0.000^1**^
				0.0006^**^
*Roseburia*				0.8709
	5.67 ± 5.74	3.76 ± 2.66	3.54 ± 2.09	0.8709
				0.8709
*Ruminococcus*				0.0024^**^
	1.32 ± 2.35	3.96 ± 2.71	1.88 ± 1.92	0.4066
				0.1407
*Phascolarctobacterium*				0.3747
	1.79 ± 1.90	0.64 ± 0.69	3.24 ± 3.25	0.5034
				0.3545

Values are presented means ± standard deviation.

*q* values were calculated using the Benjamini–Hochberg FDR method.

Pairwise comparisons were performed as follows: ET1 vs. ET2 (top), ET1 vs. ET3 (middle), and ET2 vs. ET3 (bottom).

Statistically significant differences are indicated by asterisks (^*^q < 0.05; ^**^q < 0.01).

### Analysis of association between the gut microbiota enterotypes and obesity and hyperlipidemia status following apple consumption using MaAsLin 3

To assess the impact of apple consumption on obesity status according to the gut microbiome enterotypes at the baseline, multivariable association analysis was conducted using MaAsLin 3. All variance inflation factors were <1.2, indicating stable model estimation. Exploratory analyses using time-specific PAM clustering suggested that apple consumption was associated with shifts in gut microbiota enterotype composition over the intervention period, with an increased proportion of the *Ruminococcaceae* enterotype. However, to avoid post-treatment bias, enterotype was defined at baseline for the primary longitudinal association analyses.

For analyses of obesity status, serum TG levels were included as a time-varying covariate, together with time (study visit week), sex, and gut microbiota enterotype as fixed effects. The linear interaction model was specified as follows (Model 1):


$$\begin{array}{l} \text{Microbial feature}\sim (1|\text{Subject})+\mathrm{Sex}+\mathrm{TG}+\text{Time}\\ +\text{Obesity}+\text{Enterotype}+\text{Obesity}:\text{Enterotype} \end{array}$$

**Figure 3:**
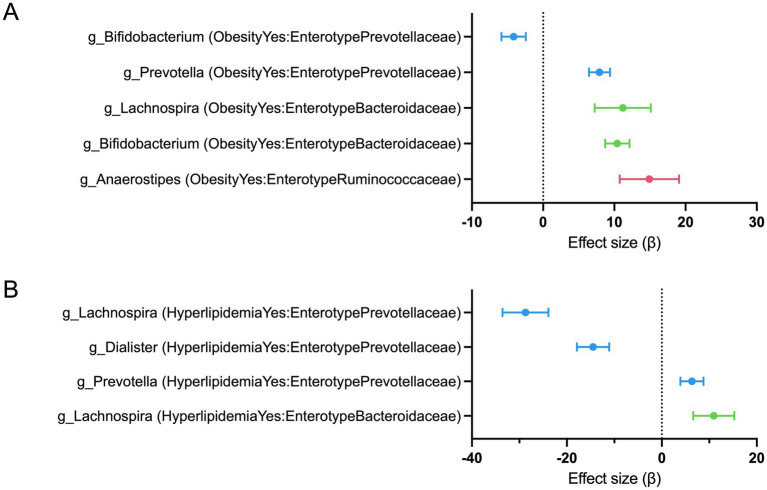
Enterotype-dependent associations between gut microbial genera and metabolic status identified using MaAsLin 3. **(A)** Interaction effects between obesity status and enterotype (Model 1). **(B)** Interaction effects between hyperlipidemia status and enterotype (Model 3). Points represent estimated regression coefficients (*β*) for each interaction term, and horizontal lines indicate 95% confidence intervals (±1.96 × SE). Only genera with FDR-adjusted *q* values <0.05 are shown.

Significant obesity × enterotype interaction terms using MaAsLin 3 were identified four microbial genera, *Bifidobacterium*, *Prevotella*, *Lachnospira*, and *Anaerostipes* (Figure 3A and Table 3). To evaluate overall associations independent of enterotype stratification, a main-effect model excluding the interaction term was also tested. In the corresponding main-effect model (Model 2), only one microbial genus, *Bifidobacterium*, showed a statistically significant association with obesity status (Table 4). Notably, Bifidobacterium is a well-characterized probiotic genus, and this significant association was therefore highlighted in the main text.

**Table 3:** Associations between gut microbial genera and obesity status, assessed using MaAsLin 3 (Model 1) with family-level PAM clustering at baseline.

	Model 1				
	(1|Subject_ID) + Sex + TG + Time + Obesity + Enterotype + Obesity:Enterotype				
Feature	Value	Name	*β* coefficient	Standard error	*q* value
*g_Bifidobacterium*	Yes:EnterotypeBacteroidaceae	ObesityYes:EnterotypePrevotellaceae	10.41	0.8744	3.57 × 10^−30^
*g_Prevotella*	Yes:EnterotypePrevotellaceae	ObesityYes:EnterotypePrevotellaceae	7.92	0.7525	1.67 × 10^−23^
*g_Anaerostipes*	Yes:EnterotypeRuminococcaceae	ObesityYes:EnterotypeRuminococcaceae	14.92	2.1288	4.80 × 10^−10^
*g_Lachnospira*	Yes:EnterotypeBacteroidaceae	ObesityYes:EnterotypeBacteroidaceae	11.19	2.0134	4.57 × 10^−6^
*g_Bifidobacterium*	Yes:EnterotypePrevotellaceae	ObesityYes:EnterotypePrevotellaceae	−4.14	0.8750	2.77 × 10^−4^

Model 1 was assessed the association of obesity status with the gut microbiome, adjusted for sex, TG, time and enterotype (covariates). Gut microbiota enterotype was classified using PAM clustering based on family-level data at baseline. *q*-values were determined by MaAsLin 3 analysis. The longitudinal data obtained at baseline and weeks 4, 8, and 12 were analyzed using linear mixed-effects models, with subject identifier included as a random effect to account for repeated measurements within individuals.

**Table 4:** Main effect model of obesity status, accessed using MaAsLin 3 (Model 2) with family-level PAM clustering at baseline.

	Model 2				
	(1|Subject_ID) + Sex + TG + Time + Obesity + Enterotype				
Feature	Value	Name	*β* coefficient	Standard error	*q* value
*g_Bifidobacterium*	Yes:EnterotypeBacteroidaceae	ObesityYes:EnterotypeBacteroidaceae	9.65	0.1619	0.00 × 10^0^

Model 2 was assessed the association of obesity status with the gut microbiome, adjusted for sex, TG, time and enterotype (covariates). Gut microbiota enterotype was classified using PAM clustering based on family-level data at baseline. *q*-values were determined by MaAsLin 3 analysis. The longitudinal data obtained at baseline and weeks 4, 8, and 12 were analyzed using main-effect model, with subject identifier included as a random effect to account for repeated measurements within individuals. *β* coefficients, standard errors, and FDR-adjusted *q* values are shown.

A parallel analysis was conducted to evaluate associations with hyperlipidemia status. The interaction model was specified as follows (Model 3):


$$\begin{array}{l} \text{Microbial feature}\sim (1|\text{Subject})+\mathrm{Sex}+\mathrm{BMI}+\text{Time}\\ +\text{Hyperlipidemia}+\text{Enterotype}+\text{Hyperlipidemia}:\text{Enterotype} \end{array}$$

MaAsLin 3 identified three microbial genera—*Prevotella*, *Dialister*, and *Lachnospira*—with significant hyperlipidemia × enterotype interaction terms (Figure 3B and Table 5). These results indicate that the associations between these genera and hyperlipidemia status differed according to enterotype. In contrast, no significant associations were identified in the corresponding main effect model for the hyperlipidemic state (Model 4).

**Table 5:** Associations between gut microbial genera and hyperlipidemia status, assessed using MaAsLin 3 (Model 3) with family-level PAM clustering at baseline.

	Model 3				
	(1|Subject_ID) + Sex + BMI + Time + Hyperlipidemia + Enterotype + Hyperlipidemia:Enterotype				
Feature	Value	Name	*β* coefficient	Standard error	*q* value
*g_Lachnospira*	Yes:EnterotypePrevotellaceae	HyperlipidemiaYes:EnterotypePrevotellaceae	−28.71	2.4715	1.24 × 10^−28^
*g_Dialister*	Yes:EnterotypePrevotellaceae	HyperlipidemiaYes:EnterotypePrevotellaceae	−14.49	1.7298	1.21 × 10^−14^
*g_Prevotella*	Yes:EnterotypePrevotellaceae	HyperlipidemiaYes:EnterotypePrevotellaceae	6.34	1.2442	6.40 × 10^−5^
*g_Lachnospira*	Yes:EnterotypeBacteroidaceae	HyperlipidemiaYes:EnterotypeBacteroidaceae	10.95	2.2117	1.18 × 10^−4^

Model 3 was assessed the association of hyperlipidemia status with the gut microbiome, adjusted for sex, BMI, time and enterotype (covariates). Gut microbiota enterotype was classified using PAM clustering based on family-level relative abundance at baseline. *q* values were determined by MaAsLin 3 analysis. The longitudinal data obtained at baseline and weeks 4, 8, and 12 were analyzed using linear mixed-effects models, with subject identifier included as a random effect to account for repeated measurements within individuals.

Additionally, to access robustness to taxonomic resolution, PAM clustering based on genus-level relative abundance at baseline was performed as a sensitivity analysis. A dominant bipartite structure was observed (*k* = 2). Furthermore, using genus-level enterotype classification, MaAsLin 3 identified *Bifidobacterium*, *Prevotella*, *Dorea*, *Lachnospira*, *Clostridium*, and *Dialister* as significantly associated with obesity status in an enterotype-dependent manner (Model 5, Supplementary Table 4), and similar associations were observed for hyperlipidemia status (Model 6, Supplementary Table 5). These finding supported the robustness of the family-level findings.

### Effects of apple consumption on lipid-related parameters based on the gut microbiota enterotype

**Figure 4:**
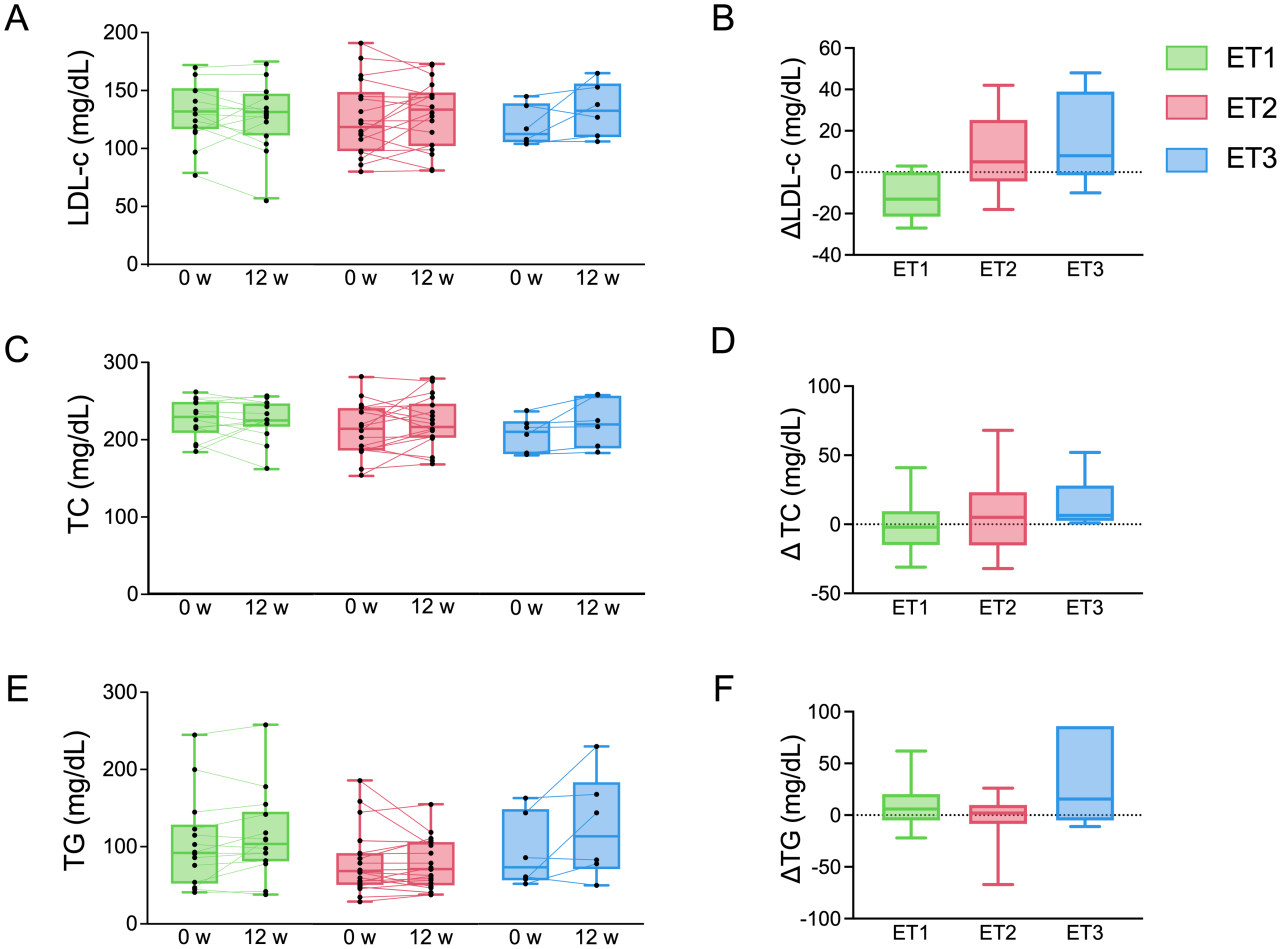
Changes in lipid-related parameters across gut microbiota enterotypes during the apple intervention. **(A,B)** LDLc, **(C,D)** TC, and **(E,F)** TG concentrations at baseline and week 12. Boxes represent the interquartile range with median; whiskers indicate minimum and maximum values. ^*^p < 0.05 and ^**^p < 0.01 by paired *t*-test; between-group differences evaluated using one-way ANOVA with Tukey’s multiple comparison test.

Overall changes in anthropometric characteristics and blood biochemical parameters during the intervention are summarized in Supplementary Table 6. Changes in lipid metabolism parameters were evaluated according to enterotype (Figure 4). While the direction of changes in lipid metabolism markers appeared to differ among enterotypes, no statistically significant differences were detected between groups.

Next, there were no significant differences in defecation frequency and Bristol Stool Form Scale scores between enterotype groups at baseline and after the intervention (Supplementary Table 7). Within the ET1 group, defecation frequency tended to decrease, while Bristol Stool Form Scale scores tended to increase following apple consumption; however, these changes were not statistically significant.

**Figure 5:**
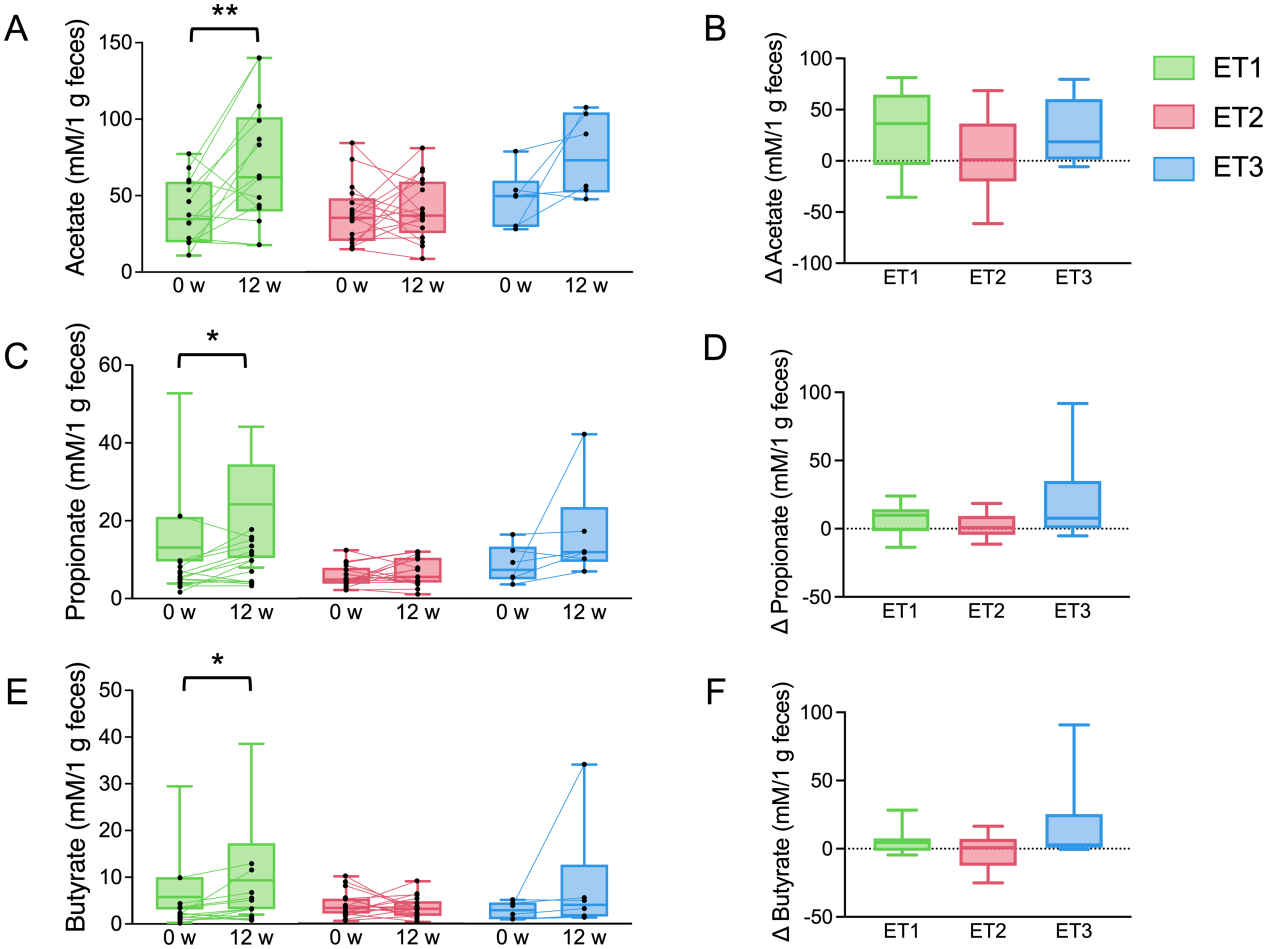
Fecal short-chain fatty acids across gut microbiota enterotypes during the intervention. **(A,B)** Acetate, **(C,D)** propionate, and **(E,F)** butyrate levels at baseline and week 12. Boxes represent the interquartile range with median; whiskers indicate minimum and maximum values. ^*^p < 0.05 and ^**^p < 0.01 by paired *t*-test; between-group differences evaluated using one-way ANOVA with Tukey’s multiple comparison test.

Additionally, changes in fecal SCFAs levels were evaluated according to enterotype (Figure 5 and Supplementary Table 8). While no significant changes were observed in ET2 or ET3, the *Bacteroidaceae*-dominant ET1 group showed a significant increase in all three major SCFAs—acetate, propionate, and butyrate—following apple consumption. Notably, these increases were not accompanied by detectable shifts in the relative abundance of representative SCFA-producing genera, *Bifidobacterium*, *Anaerostipes*, and *Lachnospira* (Supplementary Figure 3).

## Discussion

Inter-individual variability in the response to foods and their functional components is a central challenge in nutritional science and has driven increasing interest in personalized nutrition. Although apples are rich in dietary fiber and (poly)phenols and have been associated with improved metabolic health, the magnitude and consistency of their effects in human intervention studies remain variable, depending on several factors, such as the amount of fresh whole apples tested, including (poly)phenol and dietary fiber consumption, the duration of the intervention, and demographic characteristics of the subjects (23). Differences in gut microbiota composition have been proposed as a key determinant of this variability; however, enterotype-dependent responses to apple consumption, particularly in relation to obesity and lipid-related parameters, have not been well characterized.

In this 12-week intervention study, we demonstrated that the metabolic effects of daily apple consumption differed according to baseline gut microbiota structure. There were no significant differences in the glucose-related parameters. In contrast, changes in lipid-related parameters, particularly LDLc and TC, did not differ significantly between enterotype groups, SCFA levels, particularly acetate, increased significantly in participants classified as the *Bacteroidaceae*-dominant enterotype (ET1). These findings suggest that baseline gut microbiota composition may modulate specific functional responses to apple intake, particularly microbial metabolic outputs, rather than uniformly influencing host lipid profiles. In such ecosystems, primary polysaccharide degraders may create a metabolic environment that supports the activity of secondary fermenters, contributing to enhanced microbial metabolite production without marked shifts in dominant taxa. Our findings support the concept that enterotype may function as a modifier of dietary response rather than merely reflecting baseline metabolic phenotype.

The concept of gut microbiota enterotypes has been proposed as a framework to describe broad patterns of microbial community structure (3, 5). Although the stability and biological significance of enterotypes remain debated, several studies have shown that enterotype-based stratification has been applied in nutritional intervention studies to contextualize inter-individual variability in microbial and metabolic responses (25–27). In the present study, participants were classified into three enterotypes, *Bacteroidaceae*, *Ruminococcaceae*, and *Prevotellaceae*, based on baseline family-level microbiota composition. To avoid post-intervention bias, enterotypes were defined at baseline for the primary longitudinal analyses, despite exploratory analyses suggesting that apple consumption may induce gradual shifts in enterotype distribution over time.

The application of multivariable association modeling using MaAsLin 3 enabled the identification of microbial taxa associated with obesity and hyperlipidemia status after adjusting for repeated measures and relevant covariates. In the obesity status analysis, the genera *Bifidobacterium*, *Prevotella*, *Anaerostipes*, and *Lachnospira* were identified as key genera whose associations differed according to gut microbiota enterotype. These genera have been repeatedly implicated in carbohydrate fermentation and SCFA metabolism. *Bifidobacterium* is known for its capacity to degrade dietary fiber, such as pectin, and to produce acetate, which can be utilized as a substrate by other SCFA producing bacteria (41). Similarly, *Lachnospira* and *Anaerostipes* have been associated with the fermentation of complex polysaccharides and improvement of metabolic profiles in human studies. Furthermore, MaAsLin 3 analysis identified the genera *Prevotella*, and *Dialister* as enterotype-dependent taxa associated with lipid-related parameters in the hyperlipidemia status analysis. These genera have been reported to participate in carbohydrate fermentation and organic acid metabolism, suggesting the existence of alternative microbial pathways that contribute to host lipid metabolism across different enterotypes.

Notably, although genera such as *Bifidobacterium*, *Prevotella*, *Anaerostipes*, *Lachnospira*, and *Dialister* were associated with metabolic status, direct correlations between individual taxa and SCFA levels were not observed. This finding suggests that the observed functional responses may reflect community-level metabolic interactions rather than shifts in specific microbial taxa. It is noteworthy that the microbial genera associated with functional outcomes in the *Bacteroidaceae*-dominant enterotype were not limited to members of the *Bacteroidaceae* family. Rather, genera such as *Bifidobacterium* and *Lachnospira*, which belong to distinct taxonomic lineages, were identified as enterotype-associated responders. This observation suggests that enterotypes reflect dominant community structures rather than taxonomic exclusivity, and that functional responses to dietary intervention may arise from cross-feeding interactions within an enterotype-specific microbial ecosystem. Collectively, these findings indicate that baseline gut microbiota structure may influence the functional response to apple consumption, particularly SCFA production, through coordinated microbial activity. Rather than attributing metabolic outcomes to discrete enterotypes or individual taxa, the results of this study support a model in which dietary fiber intake elicits enterotype-stratified, community-level metabolic responses.

An important observation of this study was the significant increase in fecal SCFA levels, particularly acetate, in participants classified as the *Bacteroidaceae*-dominant enterotype (ET1). SCFAs are known to act as signaling molecules regulating lipid metabolism mainly through G protein coupled receptors 43 (GPR43) and 41 (GPR41) (24). However, in the present study, changes in SCFA levels were not directly associated with alterations in the relative abundance of specific bacterial genera, suggesting that these functional responses may arise from coordinated activity of the microbial community rather than from individual taxa.

In contrast, the contribution of apple polyphenols to the observed metabolic effects remains less clear. Previous human studies suggested that relatively high intake of apple (poly)phenols are required to elicit measurable lipid lowering effects, likely due to their limited bioaccessibility and bioavailability. In a human clinical study, Koutsos et al. (42), 990 mg of (poly)phenols from two apples was consumed daily, suggesting an improvement in lipid metabolism. In the present study, we used peeled and cored apples, which may contain lower (poly)phenol levels than unpeeled whole apples, the daily intake of (poly)phenols from apples was modest in comparison with the results of some earlier studies. This suggested that dietary fiber may have played a more prominent role than (poly)phenols in modulating gut microbiota and lipid metabolism. As is often the case in whole food interventions, disentangling the independent and interactive effects of dietary fiber and (poly)phenols remains challenging and warrants further investigation.

This study has several potential limitations. First, participants’ habitual diets were not strictly controlled, and intake of other (poly)phenol rich foods was not restricted, which may have introduced variability in gut microbiota composition and metabolic responses. Second, all subjects consumed peeled and cored apple flesh (300 g) containing both dietary fiber and (poly)phenols. Therefore, the specific contribution of each component could not be distinguished. Third, although outcome assessors were blinded, participants were aware of apple consumption, and expectancy effects cannot be completely excluded. Fourth, although individuals with diagnosed dyslipidemia or those receiving lipid-lowering medication were excluded, variability in baseline lipid parameters was observed, and some participants met thresholds consistent with hyperlipidemia. This metabolic heterogeneity may have influenced responsiveness to the intervention, although it may also enhance the real-world applicability of the findings. Fifth, the distribution of participants across enterotypes was unbalanced, with fewer individuals in the *Prevotellaceae*-dominant enterotype (ET3), potentially limiting statistic power in subgroup analyses. Sixth, the Greengenes 13_8 reference database used for taxonomic assignment is no longer updated, which may reduce comparability with studies based on more recent reference databases. Finally, the study employed a single-arm pre-post design without a control group. Therefore, the potential influence of temporal or seasonal variation on gut microbiota composition and metabolic outcomes cannot be fully excluded, and causal inference should be interpreted with caution.

## Conclusion

This study provides evidence that the metabolic benefits of apple consumption, particularly in lipid-related parameters, are influenced by baseline gut microbiota enterotype. The findings highlight a potential role for *Bacteroidaceae*-dominant microbiota and SCFA production in mediating these effects and underscore the importance of considering gut microbiota composition when evaluating dietary interventions. Future studies with larger sample sizes and refined dietary control will be needed to further elucidate the mechanisms underlying enterotype-dependent responses to whole foods rich in dietary fiber and (poly)phenols.

## Data Availability

The data presented in this study are publicly available. The data can be found here: https://www.ncbi.nlm.nih.gov/sra, accession PRJNA1439558.

## Supplementary material

SUPPLEMENTARY FIGURE 1 Relative abundances of the gut microbiota at phylum **(A)** and genus **(B)** levels at the baseline.

SUPPLEMENTARY FIGURE 2 Gut microbiota clusters identified at baseline based on genus-level data. **(A)** CH index for determining the optimal number of clusters. **(B)** Mean silhouette width values for each candidate cluster solution. **(C)** PCoA of genus-level relative abundance clustered using PAM, showing two clusters.

SUPPLEMENTARY FIGURE 3 Relationship the changes of SCFAs levels and the relative abundance of *Bifidobacteria* and *Lachnospira* in the *Bacteroidaceae* enterotype.

## Glossary

1.5-AG: 1.5-Anhydroglucitol
ALT: Alanine aminotransferase
AST: Aspartate aminotransferase
ASV: Amplicon sequence variants
BMI: Body mass index
BW: Body weight
CRP: C-reactive protein
CVD: Cardiovascular disease
FDR: False discovery rate
FFQ: Food Frequency Questionnaire
FPG: Fasting plasma glucose
GC-MS: Gas chromatography-mass spectrometry
γ-GTP: γ-Glutamyl transpeptidase
HDLc: High-density lipoprotein cholesterol
HbA1c: Hemoglobin A1c
LDH: Lactate dehydrogenase
LDLc: Low-density lipoprotein cholesterol
MaAsLin 3: Microbiome multivariable association with linear models 3
PAM: Partitioning around medoid
PCoA: Principal coordinate analysis
T2D: Type 2 diabetes
TG: Triacylglycerol
TC: Total cholesterol
VFA: Visceral fat area
WC: Waist circumference
