# Supplementary material for: Apple consumption is associated with obesity- and lipid-related parameters and gut microbiota profiles across enterotypes: 12-week single-blind trial in Japanese adults
Source: Front Nutr. 2026 Apr 10;13:1797920. doi: 10.3389/fnut.2026.1797920 (PMC13107531; doi:10.3389/fnut.2026.1797920)
Supplement: Supplementary file 1 [file Data_Sheet_1.PDF]

Supplementary Information

Title: Apple consumption is associated with obesity- and lipid-related parameters and gut microbiota profiles across enterotypes: 12-week single-blind trial in Japanese healthy adults

Authors: Toshihiko Shoji[1], Shiori Aoki[1], Yuki Sato[2], Mina Obara[1], Kazuaki Yoshinaga[3], Yoshiharu Takayama[1], Risa Araki[1], Saeko Masumoto[3], Tomisato Miura[2]

Supplementary Table 1. Compositions of the daily intervention 'Fuji' apples [a].

**Components**	**'Fuji' apple [b]**
Energy (kcal)[c]	159
Fat (g)[c]	0.6
Protein (g)[c]	0.3
Carbohydrate (g)[c]	46.5
Dietary fiber (g)[c]	4.2
Soluble fiber (g)[c]	1.2
Insoluble fiber (g)[c]	3.0
Hydroxycinnamic acids	
Chlorogenic acid[d]	50.79 ± 7.26
p-Coumaroylquinic acid[d]	1.49 ± 0.37
Flavan-3-ols	
(+)-Catechin[d]	1.49 ± 0.44
(−)-Epicatechin[d]	13.46 ± 2.21
Procyanidin dimers	
Procyanidin B1[e]	4.08 ± 0.97
Procyanidin B2[e]	18.39 ± 3.18
Procyanidins	
Dimers[e]	25.61 ± 4.99
Trimers[e]	11.05 ± 2.49
Tetramers[e]	16.99 ± 4.52
Pentamers[e]	19.37 ± 4.70
Hexamers[e]	15.34 ± 3.17
Heptamers[e]	15.96 ± 3.12
Polymers (over Octamer)[e]	72.39 ± 8.89
Dihydrochalcones	
Phloretin-2'-*O*-xyloglucoside[d]	2.26 ± 0.33
Phloretin-2'-*O*-glucoside[d]	3.12 ± 0.46
Flavonols	
Quercetin 3-*O*-arabinoside[d]	0.33 ± 0.10
Quercetin-3-*O*-glucoside[d]	0.38 ± 0.18
Quercetin-3-*O*-rhamnoside[d]	0.36 ± 0.09
Sum of (poly)phenols[f]	250.4 ± 36.43

The (poly)phenol content was measured according to described methods. [1,2]

[a] Data were expressed as means ± standard deviation.

[b] Consists of 300 g daily serving 'Fuji' apples (Aomori, Japan) without the core and skin.

[c] The Standard Tables of Food Composition in Japan (8th revision)

[d] Quantified in house according to Goto *et al.* [1], measurement of 10 'Fuji' apples.

[e] Quantified in house according to Obara *et al.* [2], measurement of 10 'Fuji' apples.

[f] Sum of all individual (poly)phenols (Hydroxycinnamic acids, flavan-3-ols, procyanidins, dihydrochalcones, and flavonols).

Supplementary Table 2. Baseline characteristics of the participants.

Variables	Females (n = 14)	Males (n = 24)
Anthropometrics		
Age (years)	50.93 ± 5.784	52.08 ± 6.507
Height (cm)	158.2 ± 4.617	173.0 ± 6.876
BW (kg)	54.49 ± 5.539	73.82 ± 11.00
BMI (Kg/m^2)	21.76 ± 1.821	24.58 ± 2.402
WC (cm)	74.54 ± 5.771	89.56 ± 7.830
VFA (cm^2)	46.57 ± 22.41	118.9 ± 46.50
Blood biochemistry		
Total bilirubin (mg/dL)	0.8143 ± 0.1994	0.8750 ± 0.3615
AST (U/L)	20.93 ± 5.677	25.00 ± 8.288
ALT (U/L)	16.86 ± 5.433	25.54 ± 10.99
γ-GTP (U/L)	25.71 ± 22.63	49.75 ± 44.32
TC (mg/dL)	227.9 ± 30.05	210.8 ± 28.30
TG (mg/dL)	73.64 ± 45.43	97.54 ± 51.56
Total lipids (mg/dL)	659.6 ± 91.37	641.2 ± 113.0
HDL-c (mg/dL)	78.14 ± 17.61	65.08 ± 15.20
LDL-c (mg/dL)	133.0 ± 33.06	123.2 ± 23.68
CRP (mg/dL)	0.0586 ± 0.0638	0.0754 ± 0.1252
Total bilic acid (mmol/L)	2.314 ± 1.687	3.358 ± 2.717
FPG (mg/dL)	88.86 ± 5.405	90.88 ± 11.45
HbA1c (%)	5.400 ± 0.2717	5.379 ± 0.3811
1.5-AG (μg/dL)	16.43 ± 3.976	20.67 ± 7.292
ALP (U/L)	63.21 ± 18.82	71.58 ± 20.94
LD (U/L)	186.6 ± 27.83	177.5 ± 21.23
Lifestyle		
Alcohol intake (0, 1, 2 ≧ per week) (n)	4, 4, 6	3, 3, 18
Smoking (past, present, never) (n)	0, 0, 14	10, 4, 10
Frequency of apple intake (0, 1, 2 ≧ per week) (n)	3, 2, 9	6, 8, 10
Amount of apple intake (0, 1/4, 1/2, 1 per time) (n)	3, 1, 7, 3	6, 3, 11, 4
Physical activity (0, 1, 2 ≧ per week) (n)	8, 1, 5	14, 1, 9
Defection frequency score	2.357 ± 1.008	1.792 ± 0.6580
Bristol stool form scale score	3.643 ± 1.008	3.875 ± 0.6797

Data were presented by mean ± standard deviation.
Sex was included as a covariate in multivariable analyses.
1.5- AG, 1.5-anhydroglucitol; ALP, alkaline phosphatase; ALT, alanine aminotransferase; AST, aspartate aminotransferase; BMI, body mass index; BW, body weight; CRP, C-reactive protein; FPG, fasting plasma glucose; γ-GTP, γ-glutamyl transpeptidase; HbA1c, hemoglobin A1c; HDL-c, high-density lipoprotein cholesterol; LDH, lactate dehydrogenase; LDL-c, low-density lipoprotein cholesterol; TC, total cholesterol; TG, triacylglycerol; VFA, visceral fat area; WC, waist circumference.

Supplementary Table 3. Dietary nutrient intake between before and after consumption of apple in females (n = 14) and males (n = 24) subjects.

	Females (n = 14)			Males (n = 24)		
	0 weeks	12 weeks	*P* value [a]	0 weeks	12 weeks	*P* value [a]
Energy (kcal)	1707 ± 52.47	1701 ± 35.81	0.3297	2090 ± 199.8	2119 ± 170.3	0.3846
Carbohydrate (g)	241.3 ± 10.64	241.6 ± 8.348	0.8052	276.8 ± 28.05	282.8 ± 25.42	0.1062
Fat (g)	55.25 ± 1.802	54.87 ± 1.072	0.1607	63.74 ± 6.255	63.15 ± 5.295	0.6611
Protein (g)	70.29 ± 5.703	69.35 ± 3.108	0.3143	81.60 ± 5.250	81.06 ± 5.912	0.6607
Soluble fiber (g)	3.721 ± 0.9815	3.621 ± 0.7526	0.2087	3.417 ± 0.4949	3.433 ± 0.6239	0.8115
Insoluble fiber (g)	11.86 ± 1.999	11.64 ± 1.582	0.2147	11.21 ± 1.238	11.23 ± 1.788	0.9320
Polyphenols (g)	1.079 ± 0.7480	1.034 ± 0.8626	0.8893	1.009 ± 0.3157	1.167 ± 0.4346	0.1195

Data were presented by means ± standard deviation. a, *P* values obtained female or male between pre- and post-consumption of apple tested by paired t-tests. Two-sided adjusted $P < 0.05$ was considered statistically significant.

Supplementary Table 4. Associations between gut microbial genera and obesity status, assessed using MaAsLin 3 (Model 5) with genus-level PAM clustering at baseline.

		Model 5			
		(1|Subject_ID) + Sex + TG + time + Obesity + Enterotype + Obesity:Enterotype			
feature	value	name	β coefficient	Standard Error	q-value
g_Bifidobacterium	Yes:EnterotypeBacteroides	ObesityYes:EnterotypeBacteroides	10.69	0.2846	3.72.E-306
g_Prevotella	Yes:EnterotypePrevotella	ObesityYes:EnterotypePrevotella	7.35	0.4323	1.64.E-62
g_Bifidobacterium	Yes:EnterotypePrevotella	ObesityYes:EnterotypePrevotella	-2.79	0.2846	1.95.E-20
g_Lachnospira	Yes:EnterotypePrevotella	ObesityYes:EnterotypePrevotella	-26.44	2.9386	3.07.E-17
g_Dorea	Yes:EnterotypePrevotella	ObesityYes:EnterotypePrevotella	-0.31	0.0705	7.61.E-04
g_Lachnospira	Yes:EnterotypeBacteroides	ObesityYes:EnterotypeBacteroides	10.43	2.8328	1.43.E-02
g_Clostridium	Yes:EnterotypeBacteroides	ObesityYes:EnterotypeBacteroides	1.74	0.5659	9.69.E-02

β coefficients, standard errors, and FDR-adjusted q values are shown.

Supplementary Table 5. Associations between gut microbial genera and hyperlipidemia status, assessed using MaAsLin 3 (Model 6) with genus-level PAM clustering at baseline.

Model 6					
(1|Subject_ID) + Sex + BMI + time + Hyperlipidemia + Enterotype + Hyperlipidemia:Enterotype					
feature	value	name	β coefficient	Standard Error	q-value
g_Lachnospira	Yes:EnterotypePrevotella	HyperlipidemiaYes:EnterotypePrevotella	-19.4	1.6070	5.88E-31
g_Dialister	Yes:EnterotypePrevotella	HyperlipidemiaYes:EnterotypePrevotella	-15.1	1.6052	1.16E-18

β coefficients, standard errors, and FDR-adjusted q values are shown.

Supplementary Table 6. Changes in anthropometric characteristics and blood biochemical parameters of females (n = 14) and males (n = 24) subjects during the intervention.

	Sex	0 weeks	4 weeks	8 weeks	12 weeks
Body weight (kg)	Male	73.8 ± 11.0	74.3 ± 10.9	74.1 ± 11.1	74.3 ± 11.0
	Female	54.5 ± 5.54	54.6 ± 5.61	54.9 ± 5.83	54.6 ± 5.71
BMI (kg/m^2)	Male	24.6 ± 2.41	24.5 ± 2.42	24.6 ± 2.44	24.7 ± 2.46
	Female	21.8 ± 1.83	21.8 ± 1.86	21.8 ± 1.96	21.7 ± 1.89
WC (cm)	Male	89.6 ± 7.83	90.1 ± 7.73	89.8 ± 8.36	89.9 ± 8.34
	Female	74.5 ± 5.77	77.4 ± 7.25	78.0 ± 7.34	78.9 ± 7.23
VFA (cm^2)	Male	118.9 ± 46.5	127.1 ± 46.6	125.8 ± 46.1	136.1 ± 46.2
	Female	46.6 ± 22.4	54.6 ± 24.5	52.2 ± 26.3	55.9 ± 25.1
TC (mg/dL)	Male	210.8 ± 28.3	209.6 ± 27.4	213.1 ± 29.2	215.8 ± 27.5
	Female	227.9 ± 30.05	225.3 ± 34.2	225.0 ± 32.64	234.6 ± 29.7
HDL-c (mg/dL)	Male	65.1 ± 15.2	65.3 ± 14.6	66.3 ± 16.1	64.7 ± 15.9
	Female	78.1 ± 17.6	78.5 ± 19.2	79.9 ± 21.1	79.0 ± 19.3
LDL-c (mg/dL)	Male	123.2 ± 23.7	124.0 ± 23.3	123.5 ± 25.5	127.2 ± 26.6
	Female	133.0 ± 33.1	130.6 ± 35.4	128.2 ± 32.2	134.9 ± 29.7
TG (mg/dL)	Male	97.5 ± 51.6	105.3 ± 43.6	125.3 ± 63.9	107.8 ± 53.1
	Female	73.6 ± 45.4	78.9 ± 55.5	81.0 ± 47.2	73.9 ± 42.1
Total lipid (mg/dL)	Male	641.2 ± 113.0	645.3 ± 98.7	679.3 ± 110.9	657.6 ± 88.3
	Female	659.6 ± 91.4	661.0 ± 106.4	662.6 ± 94.6	668.9 ± 86.9
FPG (mg/dL)	Male	90.9 ± 11.4	89.7 ± 7.32	90.8 ± 5.61	88.9 ± 8.10
	Female	88.9 ± 5.40	88.0 ± 6.68	89.1 ± 6.24	88.0 ± 6.25
HbA1c (%)	Male	5.38 ± 0.38	5.23 ± 0.24	5.38 ± 0.24	5.43 ± 0.31
	Female	5.40 ± 0.27	5.29 ± 0.29	5.41 ± 0.31	5.44 ± 0.24
1.5-AG (μg/dL)	Male	20.7 ± 7.29	21.6 ± 6.94	21.4 ± 6.93	21.6 ± 6.73
	Female	16.4 ± 3.98	16.4 ± 4.18	16.5 ± 4.23	16.5 ± 3.82

Data were presented by means ± standard deviation. Statistical analyses are provided for descriptive purposes only.

Supplementary Table 7. Changes of the defecation frequency and the Bristol stool form scale in the enterotype groups.

	Enterotype	0 week (baseline)		12 weeks		
			P value [a]		*P* value [a]	*P* value [b]
Defecation frequency score (Times per week)	ET1 (n=14)	1.93 ± 0.73	0.5734	1.71 ± 0.61	0.4406	0.2500
	ET2 (n=18)	2.17 ± 0.99	0.5734	2.06 ± 0.80	0.9110	>0.9999
	ET3 (n=6)	1.67 ± 0.52	0.5734	1.67 ± 0.52	0.4406	>0.9999
Bristol stool form scale score	ET1 (n=14)	3.57 ± 0.85	0.5037	3.93 ± 0.62	0.5737	0.1250
	ET2 (n=18)	3.89 ± 0.83	0.5037	4.11 ± 0.47	0.5737	0.7500
	ET3 (n=6)	4.00 ± 0.63	0.7946	4.17 ± 0.75	0.8108	>0.9999

Data were presented means ± standard deviation. a, *P* values obtained between the enterotypes by Kruskal–Wallis test, followed by Dunn's post-hoc tests with Benjamini–Hochberg adjustment (*top*, ET1 vs ET2; *middle*, ET1 vs ET3; *low*, ET2 vs ET3); b, *P* values obtained within gut microbiota enterotype between pre- and post-consumption of apple tested by paired t-tests. Two-sided adjusted $P < 0.05$ was considered statistically significant.

Supplementary Table 8. Changes of short chain fatty acids (SCFAs) levels in feces in the enterotype groups.

Short chain fatty acid (mM/1 g feces)	Enterotype	Intervention period			
		0 w	4 w	8 w	12 w
Acetic acid	ET1 (n = 14)	39.7 ± 21.0	50.2 ± 18.6	48.7 ± 33.2	70.3 ± 40.6
	ET2 (n = 17)	49.4 ± 26.0	39.6 ± 16.4	55.7 ± 39.1	55.2 ± 26.8
	ET3 (n = 6)	48.3 ± 18.5	46.1 ± 16.6	55.6 ± 23.0	76.5 ± 27.0
Propionic acid	ET1 (n = 14)	16.7 ± 11.9	15.9 ± 5.36	16.0 ± 7.92	23.3 ± 12.5
	ET2 (n = 17)	14.0 ± 6.89	11.5 ± 4.26	13.2 ± 8.12	16.8 ± 8.80
	ET3 (n = 6)	21.8 ± 12.3	20.5 ± 9.07	21.0 ± 12.5	41.9 ± 32.3
Butyric acid	ET1 (n = 14)	7.41 ± 7.22	7.88 ± 5.59	5.92 ± 2.74	12.3 ± 11.7
	ET2 (n = 17)	12.0 ± 8.26	8.05 ± 3.28	10.2 ± 9.96	10.5 ± 6.70
	ET3 (n = 6)	8.65 ± 5.45	9.01 ± 7.58	7.52 ± 3.40	25.4 ± 38.0

Data were presented means ± standard deviation. Statistical analyses are provided for descriptive purposes only.

Supplementary Figure 1

A

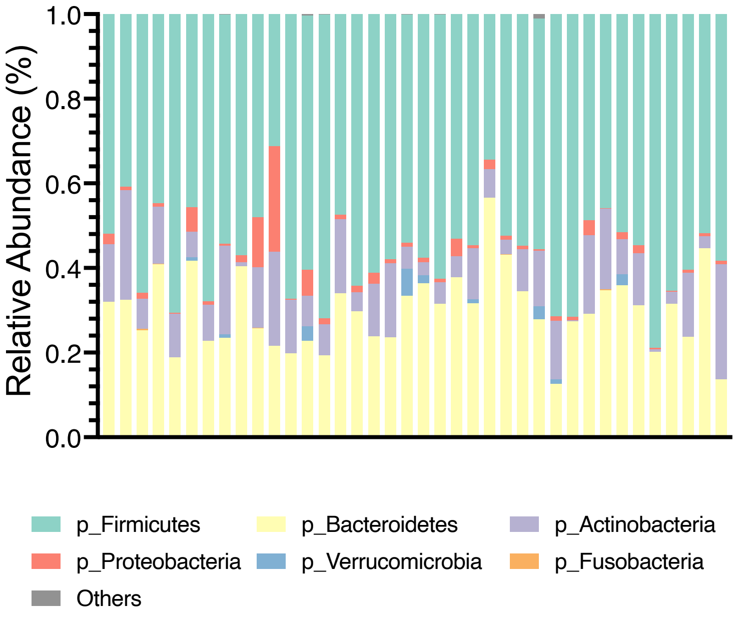

B

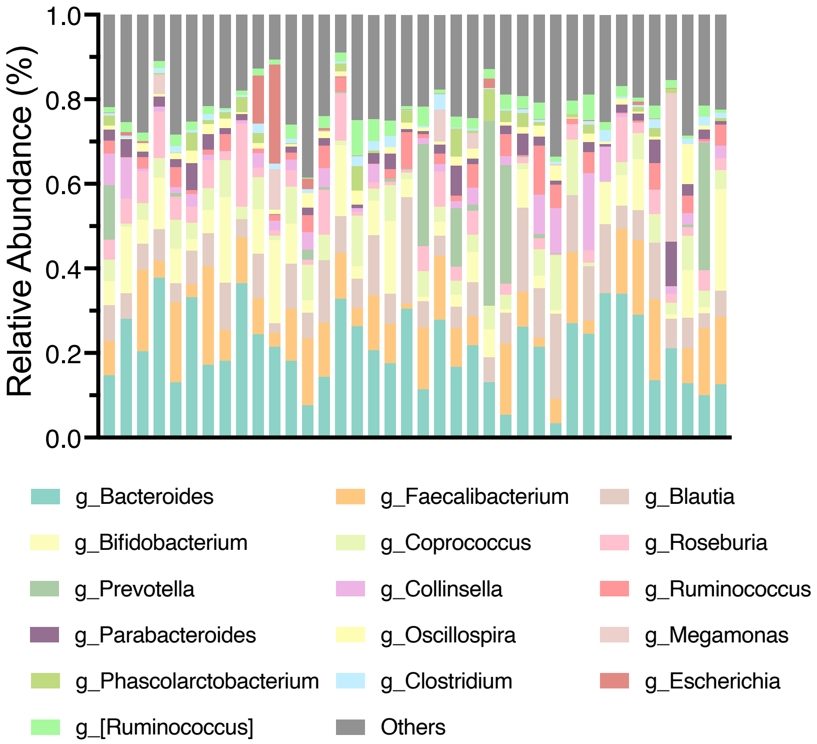

Supplementary Figure 2

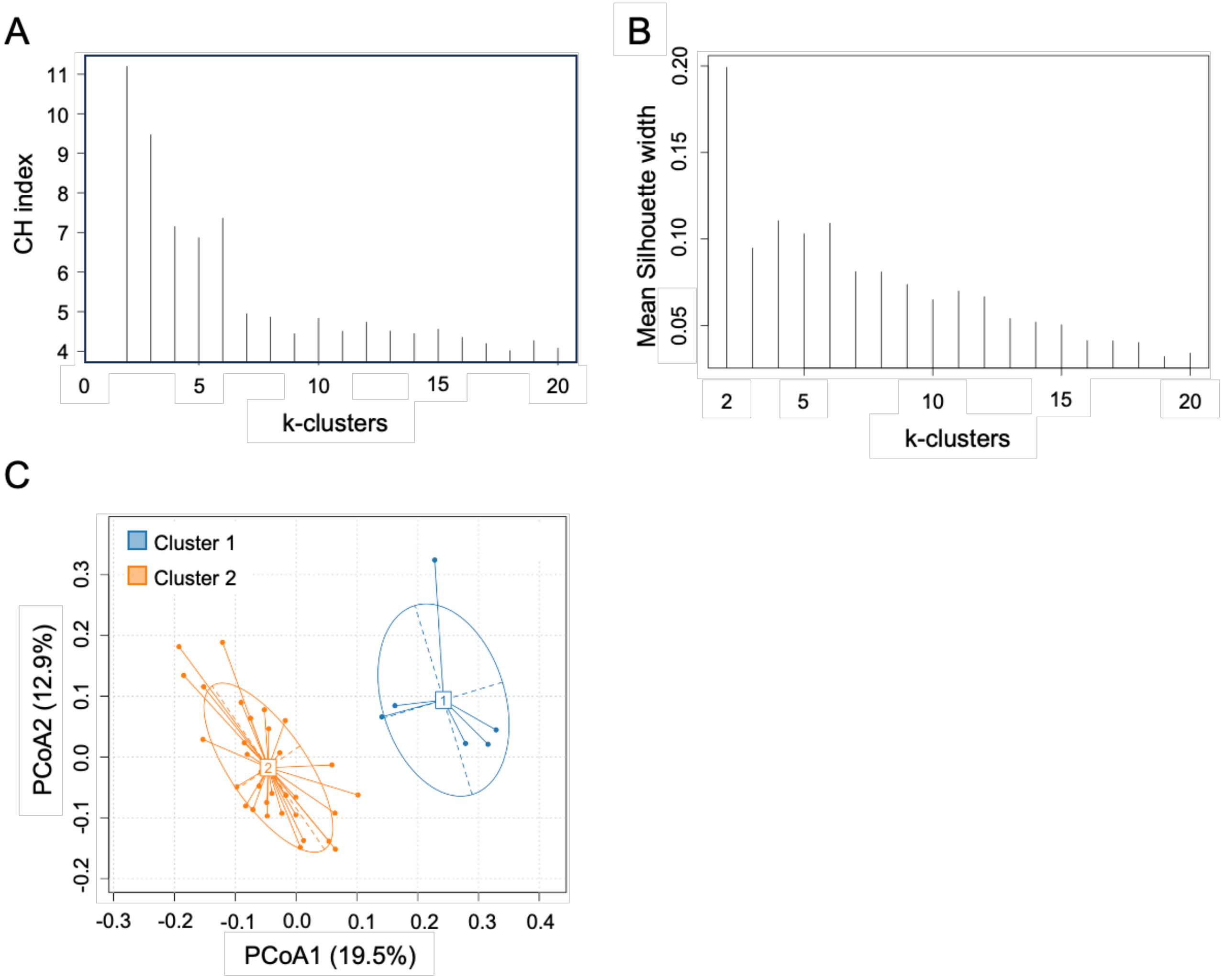

Supplementary Figure 3

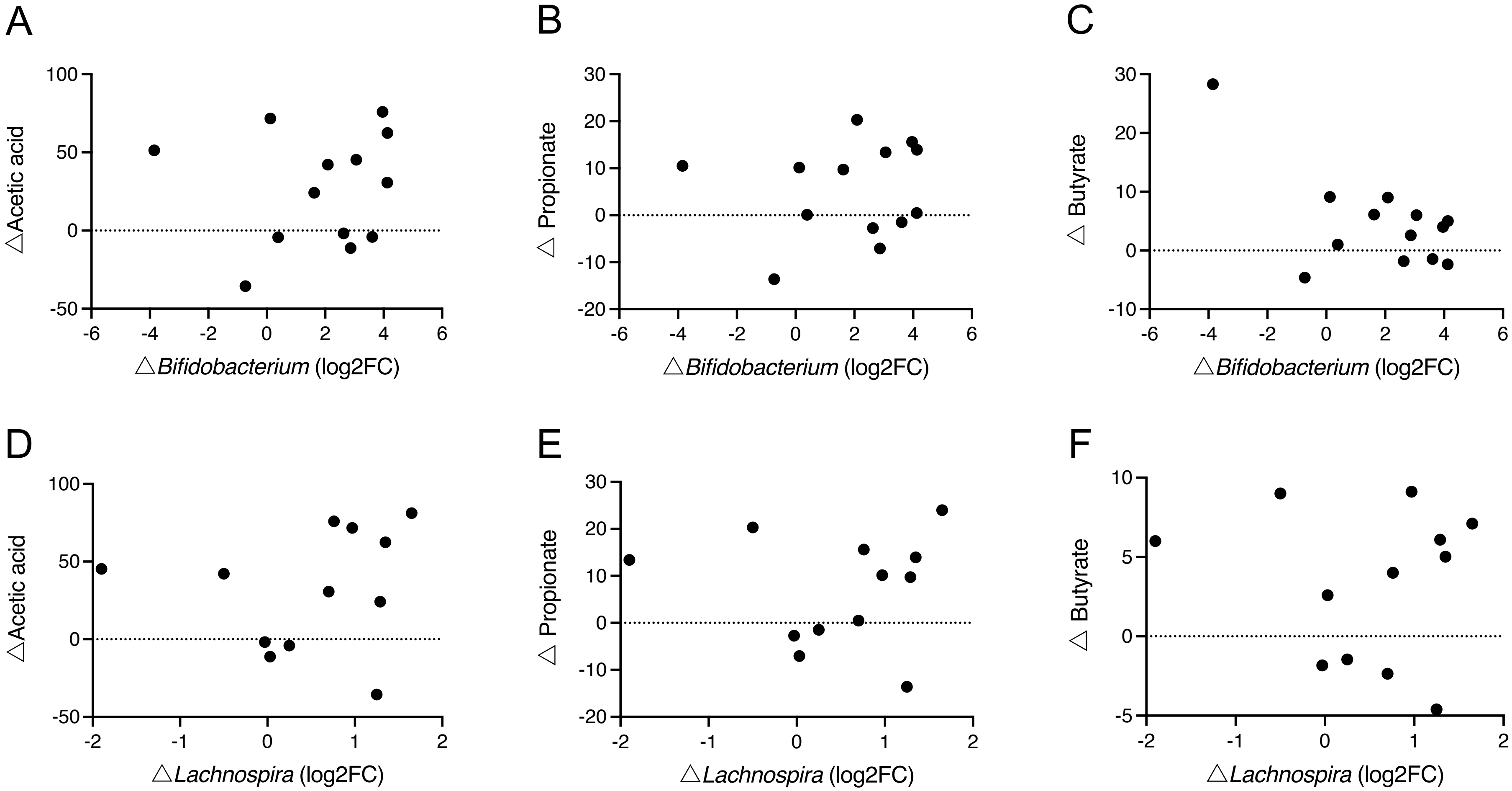

References

1 Goto T *et al.* Evaluation of polyphenolic content and potential antioxidant activity of Japanese cultivars of peaches, prunes, and plums based on reversed- and normal-phase HPLC and principal component analyses. ACS Food Sci Technol. (2021) 1:2019-2029.

2 Obara M, Masumoto S, Ono Y, Ozaki Y, Shoji T. Procyanidin concentrations and H-ORAC of apples cultivated in Japan. Food Sci Technol Res. (2016) 22:563-568.
